# HINT: High-quality protein interactomes and their applications in understanding human disease

**DOI:** 10.1186/1752-0509-6-92

**Published:** 2012-07-30

**Authors:** Jishnu Das, Haiyuan Yu

**Affiliations:** 1 Department of Biological Statistics and Computational Biology, Cornell University, Ithaca, NY 14853, USA; 2 Weill Institute for Cell and Molecular Biology, Cornell University, Ithaca, NY 14853, USA

**Keywords:** Interactomes, Networks, Protein-protein interactions, Disease

## Abstract

**Background:**

A global map of protein-protein interactions in cellular systems provides key insights into the workings of an organism. A repository of well-validated high-quality protein-protein interactions can be used in both large- and small-scale studies to generate and validate a wide range of functional hypotheses.

**Results:**

We develop HINT (http://hint.yulab.org) - a database of high-quality protein-protein interactomes for human, *Saccharomyces cerevisiae, Schizosaccharomyces pombe*, and *Oryza sativa*. These were collected from several databases and filtered both systematically and manually to remove low-quality/erroneous interactions. The resulting datasets are classified by type (binary physical interactions vs. co-complex associations) and data source (high-throughput systematic setups vs. literature-curated small-scale experiments). We find strong sociological sampling biases in literature-curated datasets of small-scale interactions. An interactome without such sampling biases was used to understand network properties of human disease-genes - hubs are unlikely to cause disease, but if they do, they usually cause multiple disorders.

**Conclusions:**

HINT is of significant interest to researchers in all fields of biology as it addresses the ubiquitous need of having a repository of high-quality protein-protein interactions. These datasets can be utilized to generate specific hypotheses about specific proteins and/or pathways, as well as analyzing global properties of cellular networks. HINT will be regularly updated and all versions will be tracked.

## Background

Numerous recent efforts in systems biology have tried to characterize the set of all possible pairwise physical interactions or the binary protein “interactome” of an organism [1-3]. Most proteins perform their functions through interactions [4]. Thus, these large-scale maps are critical in elucidating the biological roles of functional products of genes that are identified by large-scale genome and cDNA sequencing projects. Because most of these efforts are discovery-oriented and try to explore previously unknown functionalities, it is of utmost importance to ensure that the resultant maps are of high quality. Erroneous results at this stage could propagate into both ill-conceived hypotheses and futile downstream experiments. Moreover, it has been shown that high-quality interaction networks can provide key insights into fundamental topological and biological properties of cellular systems [5-8]. Although there are numerous databases [9-16] that try to systematically curate the entire repository of interactions for different organisms, there has been very little effort in filtering out unreliable ones. This has led to low overlaps between independent publications and resultant confusion as to which interactions are correct [17-19].

There are two major types of protein-protein interaction data – binary physical interactions and co-complex associations. While some databases distinguish between these two orthogonal datasets, others fail to do so. Binary interactions represent a direct biophysical interaction between two proteins. On the other hand, co-complex associations provide information about co-membership in a complex. A lot of these associations may actually represent indirect interactions [17,18]. The biological information conveyed by these two kinds of interactions is different and for many applications it is necessary to have a clear distinction between these two.

There are two major methods to obtain a global map of binary interactions – literature-curation (LC) and high-throughput experiments (HT) [18]. LC refers to systematically collecting interaction data from thousands of small-scale studies directed at validating a single or a few specific hypotheses. On the other hand, HT experiments produce large-scale interaction maps. Because most LC data are generated by hypothesis-driven experiments, it is much easier to infer biological function from those studies as compared to HT experiments. On the other hand, although the search space of some HT experiments might be focused on certain functional groups, most HT experiments are not designed to detect the presence or absence of specific interactions. Any experiment can have two kinds of bias – “assay bias” and “sampling bias”. The first arises because no assay is perfect and all experiments – HT or small-scale have their own characteristic biases [20]. However, small-scale studies also have a sampling bias, i.e., they are typically focused on one or a few proteins of interest and hence selectively sample interactions from only a part of the search space. HT experiments are free of this sampling bias, i.e., the search space is scanned without *a priori* expectations [17,19]. Thus, for many global topological analyses, it is often necessary to use only the HT datasets.

Here, we describe a publicly available protein-protein interaction database, HINT (*High-quality INT*eractomes) that directly addresses the above three issues and provides high-quality binary and co-complex interactions for human, *S. cerevisiae*, *S. pombe*, and *O. sativa.* The binary interactomes have also been divided into LC and HT subsets. Using these datasets, we show that there are significant sociological sampling biases in LC datasets, i.e., well-studied proteins tend to have more interactions in LC datasets for both human and *S. cerevisiae*. Finally, using only the high-quality HT interactions for human, we find that disease genes (i.e., genes that have a causal connection with one or more diseases) with more interactions tend to cause more diseases. Even though this result is unexpected in light of previous findings that interaction hubs are less likely to cause disease [21,22], it will help understand mechanisms of various disease processes and develop corresponding treatments.

## Results and Discussion

### Data source for protein-protein interactions

The set of all protein-protein interactions for the organisms was downloaded from the public databases – BioGrid [9], DIP [10], HPRD [11], IntAct [12], iRefWeb [13], MINT [14], MIPS [15] and VisAnt [16]. Not all four organisms were present in all the databases. Though some of the databases mentioned above store both genetic and physical interactions, only physical interactions were used in building the interactomes. Certain tools [13,23] also provide scoring schemes for protein-protein interactions. However, we do not include these for HINT as they integrate both computational predictions and experimentally determined interactions. Our goal is to provide a repository of only experimentally well-validated high-quality protein-protein interactions.

### Building the database

**Figure 1:**
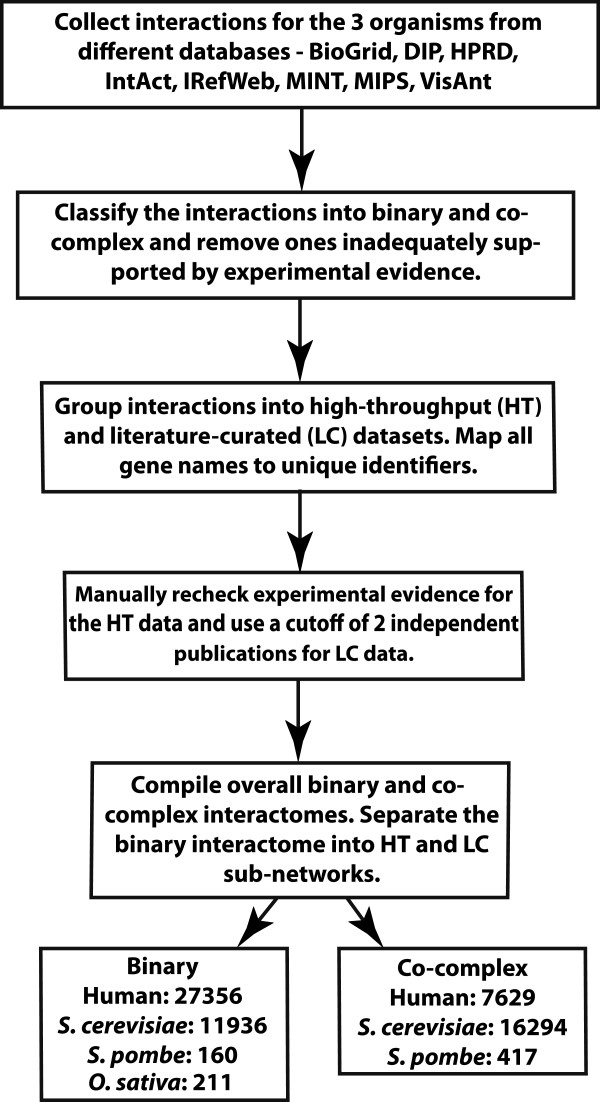
Flow diagram depicting the series of steps used to build HINT.

Figure 1 summarizes how HINT was built. For each organism and each source database, a filter was applied to generate non-redundant lists of appropriate interactions for the two categories – binary and co-complex. The filter classifies interactions into the correct groups and removes ones that are inadequately supported by experimental evidence. The binary interactions were further classified as HT and LC based on the nature of the experiments that produced them. If the experiment in support of the interactions discovers greater than a cutoff number of interactions, it is classified as HT. To determine the cutoff, we calculated the distribution of number of interactions reported by each unique publication. The cutoff (> = 100 interactions) corresponds to the top 0.5 percentile of studies, when all publications are ranked in decreasing order of interactions reported per study (Additional file 1: Figure S1). For co-complex associations, there exists no such clear distinction between HT and LC because the average number of interactions detected in a single experiment is significantly higher.

The next step was to remove low-quality interactions. For ones supported by HT publications, a non-redundant list of papers was compiled and each publication was manually examined to verify that the actual experiments used by the authors agree with the evidence codes cited by the curators. All papers for which there was an error in this matching process were removed. Moreover, papers that do not validate the interactions obtained were also not included in HINT. Although some HT affinity purification followed by mass spectrometry (AP/MS) experiments producing co-complex associations report confidence scores, most binary HT experiments do not. For co-complex interactions, we require all interactions to be reported by two papers or more to ensure quality. For HT binary experiments, some report datasets of different levels of confidence – usually, core vs. non-core. We always include the highest-quality dataset (i.e., the core set only). Moreover, we ensure that every single interaction included is high-quality (please see Quality control section). Within this high-quality dataset, the users of HINT are free to choose their own confidence cutoff based on any combination of the number of supporting publications and evidence code. For LC interactions, it is not possible to replicate this process, as the number of papers is too high. It has been shown that a large fraction of the LC interactions supported by a single publication cannot be verified [18,24]. Curation is an extremely painstaking process and we acknowledge that there may be some high-quality interactions supported by only one publication. However, it is impossible to distinguish them from the larger fraction that has been demonstrated to be of lower quality/erroneous [17,19]. Our goal here is to present to the community only a high-quality dataset that is free of potential biases due to differential curation of the same source publication. Only those LC interactions that are supported by two or more publications are preserved in our database. Table 1 provides a summary of the source databases used (version and download date). Table 2 reports the number of high-quality interactions in each of these databases in each category (no high-quality co-complex associations were obtained for *O. sativa*). 

**Table 1:** Source databases – download dates and versions

**Database**	**Download date (version if applicable)**
BioGrid	11 January 2012 (v 3.1.84)
DIP	12 January 2012
HPRD	12 January 2012 (Release 9)
IntAct	12 January 2012 (2011 release)
iRefWeb	12 January 2012 (v 4.1)
MINT	17 January 2012 (2012 release)
MIPS	17 January 2012
VisAnt	13 January 2012 (Release 3.93)

**Table 2:** Database statistics – Summary of high-quality interactions obtained from the different data-sources and those finally included in HINT

**Database**	**Human binary**	**Human co-complex**	***S. cerevisiae *binary**	***S. cerevisiae *co-complex**	***S. pombe *binary**	***S. pombe *co-complex**	***O. sativa *binary**
HINT	27356	7629	11936	16294	160	417	211
BioGrid	13244	6175	8458	13053	110	375	-
DIP	455	336	1905	1510	-	-	
HPRD	15449	-	-	-	-	-	-
IntAct	10226	2438	4906	10539	72	201	1
iRefWeb	17216	5554	10005	14122	125	386	-
MINT	2379	740	1397	1603	-	-	-
MIPS	-	-	1624	274	-	-	-
VisAnt	13913	4882	7337	13632	69	264	210

For the binary network, we generated two sub-interactomes – the high-quality LC (HQ-LC) and the high-quality HT (HQ-HT) sub-interactomes. Interactions that are supported by both forms of evidence belong to both.

Tables 3, 4 and 5 provides summary statistics for the different interactomes and their sub-classes. The numbers refer to unique entries and any interaction validated in multiple orientations (e.g., bait and prey in binary interaction detection experiments) or by different research groups is counted as a single entity. We find that the average degree for both *S. pombe* and *O. sativa* are much lower than that of human or *S. cerevisiae* for both binary and co-complex data. This shows that the *S. pombe* and *O. sativa* interactomes are still mostly unexplored. There is also a sharp increase in the average degree from binary to co-complex for *S. cerevisiae*. This is expected given that models to generate topologies of co-complex networks tend to include several or all possible combinations [25]. However, the same does not hold true for human. This probably indicates that the human co-complex interactome is underexplored as compared to the *S. cerevisiae* one. 

**Table 3:** Network statistics for binary interactions

**Organism**	**Unfiltered binary interactions**	**Filtered binary interactions**	**No. of proteins in filtered network**	**Average degree of filtered network**
Human	57989	27356	8256	6.629
*S. cerevisiae*	18973	11936	3709	6.436
*S. pombe*	1059	160	174	1.839
*O. sativa*	229	211	222	1.901

**Table 4:** Network statistics for co-complex interactions

**Organism**	**Unfiltered co-complex interactions**	**Filtered co-complex interactions**	**No. of proteins in filtered network**	**Average degree of filtered network**
Human	55452	7629	3189	4.785
*S. cerevisiae*	99343	16294	3380	9.641
*S. pombe*	3424	417	389	2.144

**Table 5:** HT and LC interactions

**Organism**	**Number of HT interactions**	**Number of LC interactions**
Human	20187	8732
*S. cerevisiae*	9876	3624

Note: There are no high-quality HT experiments for *S. pombe*. Hence the whole interactome is LC. There are no high-quality LC experiments for *O. sativa*. Hence the whole interactome is HT.

**Figure 2:**
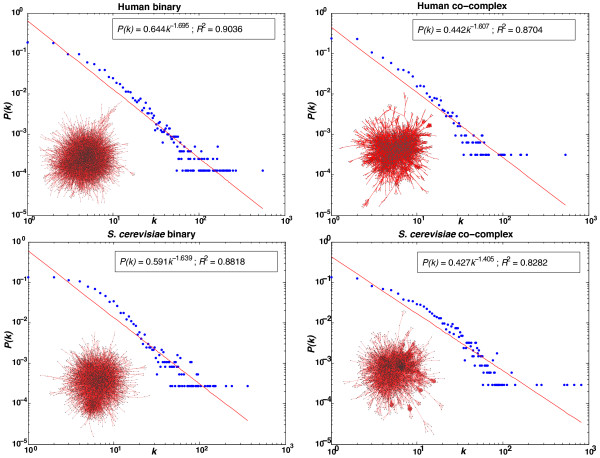
**Binary and co-complex interactomes and degree distribution plots for human and *S. cerevisiae.***

Figure 2 depicts the binary and co-complex interactomes for human and *S. cerevisiae*. The degree distribution of each of the networks is also illustrated and these plots correspond well with the theoretical expectation of the networks being scale-free [26]. It is not possible to produce these plots for *S. pombe* and *O. sativa* as the interactomes for these organisms are severely underexplored. The *S. pombe* networks are available as Additional file 2: Figure S2. 

### Quality control

There has been a great deal of effort in the literature at discovering new protein-protein interactions in different species to gain an understanding of the entire interactome of that organism. However, due to experimental errors and inaccurate curation, databases often contain interactions that are low quality/erroneous [18]. Since accuracy is of paramount importance in generating new hypotheses using these interaction data, it is essential to have an easily accessible repository of high-quality binary protein-protein interactions. HINT is a repository created by combining information from commonly used databases. To ensure quality control, we adopt the following protocol. Since the number of HT publications is relatively low as compared to the vast number of small-scale studies, we manually inspect each of the HT studies (Additional file 3: Table S1 and Additional file 4: Table S2). We ensure that high-quality HT experiments included in HINT have been verified by orthogonal traditional assays (e.g., co-immunoprecipitation). Some experiments that do not perform any validation of their screen are considered low-quality and therefore removed. More recently, we developed a statistical framework to comprehensively evaluate the quality of HT datasets verified by orthogonal assays in both human and *S. cerevisiae*[17,19]. Using this framework, we can quantitatively and experimentally measure the quality of individual interactions, as well as the whole dataset. The quality of interactions reported by a HT experiment can be measured by two independent statistical parameters – the number of interactions validated, i.e., the “validation rate” and the number of interactions that could be re-tested in the validation carried out, i.e., the “retest rate”. The first parameter is a measure of the confidence associated with the validation carried out (i.e., more confidence can be associated with the results when a larger fraction of the reported interactions are validated), while the second one directly assays the reproducibility of the HT experiment. We carried out a comprehensive re-curation for all HT experiments included in HINT. A list of these parameters for all the HT experiments can be found in Additional file 5: Table S3 and Additional file 6: Table S4.

On the other hand, since it is impossible to manually check all small-scale studies, we require two independent publications to report the same interaction for it to be included in our dataset. This is because while some interactions from dedicated small-scale studies are high-quality and have been repeated multiple times in the literature, a significant fraction of interactions from small-scale experiments are not easily reproducible. In fact, many of the interactions that cannot be reproduced are supported by only one publication, were not produced by dedicated experiments and were often not even mentioned in the paper (Additional file 7: Figure S3) [18]. More importantly, it has been experimentally shown that such interactions are indeed of low quality [17,19]. Thus, our repository of high-quality interactions contains only manually validated HT experiments and interactions from small-scale studies that have been reported at least twice in the literature.

**Figure 3:**
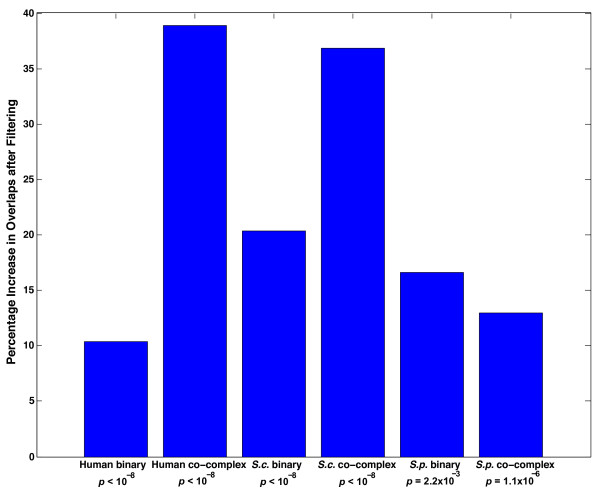
**Average overlap percentage between all pairs of databases for binary and co-complex interactions in human, *S. cerevisiae *(*S.c.*), and *S. pombe *(*S.p.*) before and after filtering.**

To further validate the filtering approach used we adopted the following method. For each organism and interaction type, percentage overlaps between all pairs of databases that contain data relevant to that category were calculated before and after filtering. Since all these databases are curating the same information, we would expect the overlaps between any two of them to be high. However, that is not the case and we find low overlaps between pairs of databases. This supports our hypothesis that some of the information contained in these datasets is low-quality/incorrect. However, if our filtering scheme successfully removes these low-quality/incorrect interactions, the pairwise overlap between databases should increase considerably after filtering. We find that this is indeed the case. For each organism and interaction type, there is a significant enrichment in the average pairwise overlap between databases after filtering (Figure 3; *P*-values calculated using a cumulative binomial test). Specifically, let the maximum number of interactions for a certain organism and interaction type that can be common to a particular database pair before and after filtering be denoted by *Mb*_*i*_ and *Ma*_*i*_ respectively, where *i* is an index to denote the database pair. Let the percentage overlaps before and after filtering for that pair be denoted by *Pb*_*i*_ and *Pa*_*i*_ respectively. The average percentage overlap for that organism and interaction type before (*AvPB*) and after filtering (*AvPA*) are calculated as:

$$AvPB=\frac{\sum_{i}Mb_{i}\;x\;Pb_{i}}{\sum_{i}Mb_{i}}$$

$$AvPA=\frac{\sum_{i}Ma_{i}\;x\;Pa_{i}}{\sum_{i}Ma_{i}}$$

### Querying the database

**Figure 4:**
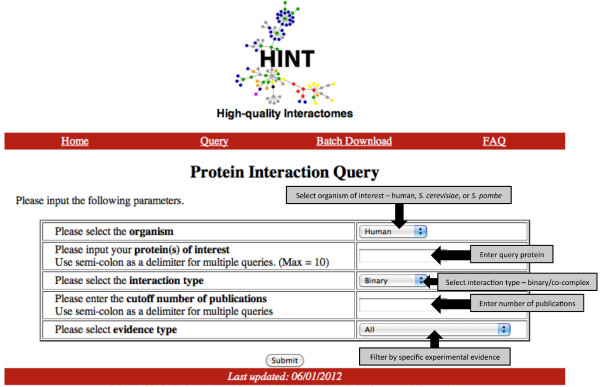
Screenshot of the user interface of HINT.

The database has two major parts – a query interface and a batch download for the entire interactomes of the organisms. Figure 4 illustrates the user interface of HINT. The pooled interactions can be queried in the following manner.

The organism of interest is selected from a drop-down menu followed by entering the query proteins separated by semi-colons. Up to a maximum of 10 proteins can be entered per query. The database supports Entrez gene IDs [27] and gene names for proteins in human, ORF names and gene names for proteins in *S. cerevisiae*[28] and *S. pombe*[29] and Uniprot ids for *O. sativa*[30]*.* The user also has the option of specifying the cutoff number of publications for each of the query proteins. One can also specify a particular evidence type for searching interactions. For each interacting protein, the gene name is listed in the first column followed by the list of Pubmed IDs of the papers supporting this interaction in column 2. The last column lists the PSI-MI evidence code [31] that describes the kind of evidence supporting the interaction. The gene names are linked to the NCBI Entrez Gene database [27] for human and *S. cerevisiae*, the GeneDB database [32] for *S. pombe*, and the Uniprot database [30] for *O. sativa*. The PubMed IDs link to the NCBI website for the relevant abstracts.

For batch download, separate links are provided for binary and co-complex interactomes for each organism. The binary interactome is also divided into the LC and HT networks. One notes here that the LC and HT networks are not completely mutually exclusive. There are certain protein-protein interactions that have been discovered both by HT experiments and by LC. There are included in both interactomes.

Using HINT, it will now be possible to analyze, visualize, and generate reliable hypotheses about a part of or the complete interactome of the four different organisms – human, *S. cerevisiae*, *S. pombe*, and *O. sativa.* Future efforts may be directed at similarly collecting and filtering data for other organisms and also updating the current dataset based on new findings.

### Binary vs co-complex

HINT clearly distinguishes between binary and co-complex interactions. The binary network represents direct interactions between two proteins. On the other hand, the co-complex network merely indicates membership of a group and does not necessarily imply pairwise interactions. In most cases, the exactly topology of the complex is unknown. Two primary methods – the spoke model and the matrix model are used to represent these complexes. However, both models are approximations and merely suggest possible topologies [25]. Since different reports base their choice of model on study-specific conditions, all co-complex associations were included as curated in the source databases. No re-curation was performed. Moreover, compared to co-complex interactome models, binary maps have a greater fraction of transient signaling connections and inter-complex connections [17,33]. Since these two datasets represent fundamentally different biological entities, their overlap is low (Additional file 8: Figure S4) and it is important to differentiate between them in certain studies. For example, recent studies have examined how mutations may either lead to complete loss of gene products or edge-specific changes in the interactome [34,35]. We show in a recent study that the pathogenesis of human disease can be better understood by looking at the position of mutations on interaction interfaces [36]. These approaches are applicable to direct binary interactions, as it is more difficult to infer interface pairs from co-complex associations. The latter can be resolved using information on three-dimensional structures of protein complexes if these are available. Thus, based on the context, it may be more appropriate to use one interactome over the other. Moreover, there are significant differences in the topological properties of these two networks. We calculated the clustering coefficient [37] and the edge betweenness [38] for the different interaction networks in HINT. Clustering coefficient measures the density of clustering in an interaction network [37]. We find that co-complex networks have a significantly higher clustering coefficient (*P* < 10^-8^ in both cases as calculated by a two-sample Kolmogorov-Smirnov test) than binary networks (Additional file 9: Figure S5). This shows that co-complex associations tend to be much more dense in terms of topological structure. Edge betweenness is used to detect community structure in networks. A higher betweenness value for an edge indicates that it connects different modules and disrupting this edge will fragment the network into disjoint components [38]. We find that binary networks for both human and *S. cerevisiae* have a significantly higher betweenness (*P* < 10^-8^ in both cases as calculated by a two-sample Kolmogorov-Smirnov test) than co-complex networks for the two organisms (Additional file 9: Figure S5). This suggests that co-complex associations form tightly regulated modules and binary interactions are often used to form links between these modules. We did not use the *S. pombe* or *O. sativa* networks for our global topological calculations as these interactomes are highly underexplored at this stage and the small number of interactions available make the networks unsuitable for global analyses.

### HT protein-protein interactions in understanding human disease

People have realized in the last decade that a human disease is rarely the consequence of an isolated abnormality in a particular gene but is generally the outcome of complex perturbations of the underlying cellular network [39]. This has led to systematic studies of interactome networks and numerous insights have been obtained from such studies. The structure of these networks is governed by key biological principles and changes in their global properties may be linked to human disease [40]. Further advances in such studies are expected to uncover the biological significance of disease-associated mutations discovered by genome-wide association studies [41] and help in identifying biomarkers and novel drug targets [39].

**Figure 5:**
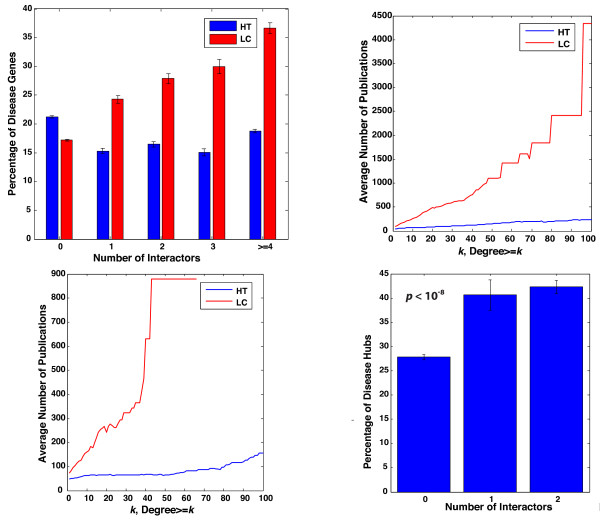
**A. Percentage of disease genes within proteins that have 0, 1, 2, 3, and > =4 interactions respectively. B**. Plot of average number of publications associated with a protein versus the cumulative degree of the protein in the HT and LC interaction networks in human. **C**. Plot of average number of publications associated with a protein versus the cumulative degree of the protein in the HT and LC interaction networks in *S. cerevisiae*. **D**. Percentage of disease hubs within disease genes that have 0,1, and > =2 interactions respectively.

Previous studies have shown that protein hubs tend to be essential genes [42,43]. Therefore, one interesting question is whether a lot of the hubs are disease genes. Using the HT interactome, we examined the distribution of disease genes across number of protein-protein interactions. We found that disease-genes tend not to be hubs (Figure 5A; error bars correspond to standard error of the mean assuming a binomial distribution). This result is consistent with earlier studies that find that disease genes are usually non-essential and occupy peripheral positions in the human interactome [21,22]. The finding is logical in light of an evolutionary argument – for essential genes, mutations would be more likely to affect fitness to the extent of causing embryonic lethality [21,22]. 

However, we were unable to reproduce the same results using the LC interactome (Figure 5A; error bars correspond to standard error of the mean assuming a binomial distribution). There is a significant increase (*P* < 10^-8^ as calculated by a one-way ANOVA) of percentage of disease genes with degree for proteins that have at least one interaction. This led us to believe that the difference could be due to study biases in the LC data. To systematically analyze if this is true, we plotted the average number of publications against the number of interactions of proteins separately for the HT and LC interactomes. Intuitively, there should be no strong correlation between these two entities as the number of publications associated with a protein should have no connection with its degree. The average number of publications does not vary significantly with degree for the HT dataset but increases dramatically for the LC interactome (see Figures 5B and 5 C). This illustrates the strong study bias in the LC data – proteins with a greater number of interactions tend to be revisited more often by small-scale studies. Our results are consistent with earlier findings that the degree of proteins in the LC interactome is strongly correlated with the number of publications associated with them [17,44]. This makes the LC interactome unsuitable for global topological analyses. The low overlap between the HT and LC interactomes (Additional file 9: Figure S4) also confirms that these are in fact two separate networks that need to be appropriately used based on the context.

To further investigate whether protein interactomes can help us understand disease mechanisms and uncover previously unknown disease genes, we used the HT human interactome to analyze what fraction of disease genes are disease-hubs, i.e., genes causing multiple diseases. We examined the distribution of disease-hubs as a function of their degrees (Figure 5D; error bars correspond to standard error of the mean assuming a binomial distribution). We observed that proteins with a higher number of interactions are significantly more likely to be disease hubs (*P* < 10^-8^ as calculated by a one-way ANOVA). Though this may seem contradictory to earlier findings in Figure 5A, these two are in fact independent results. It is true that if a disease gene has more interactions, there is a higher probability of its fitness being affected. However, in Figure 5D, we focused only on disease genes. By virtue of the fact that these are observed in the population as disease genes, their mutations are less likely to cause embryonic lethality. Therefore the evolutionary constrains in Figure 5A do not apply here. It is logical to expect that a disease protein with multiple interactions will have a greater propensity for causing multiple diseases. This is because a protein with more interactions is involved in more biological functions [42]. This result also means that protein-protein interactions are important in the pathogenesis of many human diseases. Further studies on alteration of interactions by disease mutations may reveal insights into molecular mechanisms of various diseases and provide information about potential drug targets.

## Conclusions

HINT is a comprehensive repository of high-quality binary and co-complex physical interactions in human, *S. cerevisiae*, *S. pombe*, and *O. sativa.* It establishes and implements systematic techniques for separating interactions based on both type (i.e., binary and co-complex) and data-source (i.e., LC and HT). Making these distinctions is critical for many applications. Using only the HT dataset, we demonstrated that human disease genes with a greater number of interactions tend to cause more diseases. Future directions involve implementation of the same techniques for other organisms of biological interest.

## Methods

### Evidence codes and ID-mapping

As one of the primary goals of the database is to clearly distinguish binary interactions from co-complex associations, two separate and mutually exclusive lists of evidence codes were created – one for each category. An evidence code is a unique number assigned by the PSI-MI initiative to a particular form of experimental information in support of an interaction [31]. The lists used for both categories can be found in Additional file 10: Table S5, Additional file 11: Table S6 and Additional file 12: Table S7. Using these lists, all the interactions were classified into binary and/or co-complex. Interactions supported by evidence codes that are in neither of the two lists are excluded. Different databases use different gene identifiers and as this may lead to error, all gene identifiers in each database were converted to Entrez gene IDs for human, ORF names for *S. cerevisiae* and *S. pombe*, and Uniprot ids for *O. sativa*. For each of the organisms, gene names (when available) are also provided in the bulk download files. Mapping files we obtained from Uniprot [30] and the NCBI gene databases.

As described earlier, for an interaction to qualify as high-quality, it has to have at least one manually verified HT evidence code or at least two LC evidence codes supporting it. For certain database-specific details, please refer to the Methods section in the Supplementary information (Additional file 13: Supplementary Methods).

### Protein-protein interactions and human disease genes

To look at the distribution of human disease genes across number of protein-protein interactions, the following protocol was used. The total number of human proteins is taken to be 20,000. For the LC and the HT interactomes, we separately calculated the number of proteins that take part in 1, 2, 3, and > =4 interactions respectively. The difference of 20,000 and the sum of proteins in all these categories represents the number of proteins that have no known interactions in that particular network. Thus we have the number of proteins with 0, 1, 2, 3, and > =4 interactions for both interactomes. The mapping between human genes and diseases is obtained from OMIM [45] and HGMD [46].Then the following formula was used to calculate the percentage of disease genes in each category (*PG*_*i*_):

$$PG_{i}=\frac{N_{i}\;x\;100_{{}_{}}}{T_{i}}$$

where Ni is the number of disease genes in bin i and Ti is the total number of genes in bin i.

Here each bin corresponds to the number of interactions – 0, 1, 2, 3, and > =4 respectively. These values have been shown in Figure 5A. The error bars represent standard error of the mean assuming a binomial distribution (each gene is either involved or not involved in disease).

To calculate the sub-percentage of disease hubs in each category (*PH*_*j*_), the following formula was used:

$$PH_{j}=\frac{N_{j}\times 100}{T_{j}}$$

where Nj is the number of disease hubs in bin j and Tj is the total number of disease genes in bin j.

Here each bin corresponds to the number of interactions – 0, 1, and > =2 respectively and a disease hub is any disease gene implicated in three or more diseases. These values have been shown in Figure 5D. The error bars represent standard error of the mean assuming a binomial distribution (each protein is either a disease hub or it is not).

## Abbreviations

AP/MS: Affinity Purification followed by Mass Spectrometry; HQ-HT: High-Quality High-Throughput; HQ-LC: High-Quality Literature-Curated; HT: High-Throughput; LC: Literature-Curated; ORF: Open Reading Frame; PSI-MI: Protein Standards Initiative Molecular Interaction.

## Competing interests

The authors declare that they have no competing interests.

## Author contributions

HY conceived the study. JD built the database and performed all the calculations. JD and HY interpreted the results and wrote the manuscript. All authors read and approved of the manuscript in its final form.

## Supplementary Material

Additional file 1 Histogram of number of interactions reported by different studies focusing on detecting binary protein interactions in human and *S. cerevisiae* respectively. Click here for file

Additional file 2 Binary and co-complex interaction networks in *S. pombe*. Click here for file

Additional file 3 Binary protein-protein interactions in human – HT studies. Click here for file

Additional file 4 **Binary protein-protein interactions in *S. cerevisiae *– HT studies.** Click here for file

Additional file 5 Validation and retest rates for binary protein-protein interactions in human – HT studies. Click here for file

Additional file 6 Validation and retest rates for binary protein-protein interactions in *S. cerevisiae* – HT studies. Click here for file

Additional file 7 Example of an interaction from a small-scale study with low-quality supporting evidence. Click here for file

Additional file 8 Overlaps between binary and co-complex interaction, HT and LC interaction networks in human and *S. cerevisiae*. Click here for file

Additional file 9 Clustering coefficient and edge betweenness for binary and co-complex networks in human and *S. cerevisiae*. Click here for file

Additional file 10 List of PSI-MI evidence codes used to classify binary interactions and co-complex associations. Click here for file

Additional file 11 Mapping used to convert MIPS evidence codes to PSI-MI evidence codes. Click here for file

Additional file 12 Mapping used to convert VisAnt evidence codes to PSI-MI evidence codes. Click here for file

Additional file 13 Description of database-specific filtering techniques. Click here for file
