# Supplementary material for: HINT: High-quality protein interactomes and their applications in understanding human disease
Source: BMC Syst Biol. 2012 Jul 30;6:92. doi: 10.1186/1752-0509-6-92 (PMC3483187; doi:10.1186/1752-0509-6-92)
Supplement: Additional file 1 — Histogram of number of interactions reported by different studies focusing on detecting binary protein interactions in human and S. cerevisiae respectively. [file 1752-0509-6-92-S1.pdf]

Supplementary Methods

Binary interactions

The databases MIPS [1] and VisAnt [2] do not follow standard PSI-MI [3] evidence codes. For these databases, the native evidence codes were mapped to PSI-MI codes using Supplementary Tables 2 and 3 respectively.

Co-complex associations

The databases MIPS [1] and VisAnt [2] do not follow standard PSI-MI evidence codes [3]. For these databases, the native evidence codes were mapped to PSI-MI codes using Supplementary Tables 2 and 3 respectively. Since there is no distinction between LC and HT for co-complex associations, a uniform cutoff of two or more independent publications was used for any interaction to be included in our interactome. A new assay called DHFR-PCA reported by Tarassov et al [4] was included with evidence code 9999 as it does not have any corresponding PSI-MI evidence code.

Reasoning for quality control procedure adopted

1) Since, the number of HT publications is relatively low as compared to the vast number of small-scale studies, we manually inspect each of the HT studies to ensure their correctness. We make sure that all high-quality HT experiments included in HINT have been verified by orthogonal traditional assays.

2) More recently, we developed a statistical framework to comprehensively evaluate the quality of HT datasets verified by orthogonal assays in both human and *S. cerevisiae* [5, 6]. Using this framework, we can quantitatively and experimentally measure the quality of individual interactions, as well as the whole dataset.

3) There are two separate statistical parameters that can be used to assess the quality of validation – the number of interactions validated in a HT experiment, i.e., the "validation rate" and the number of interactions that could be re-tested in the validation carried out, i.e., the "retest rate". The first parameter is a measure of the confidence associated with the validation carried out (i.e., more confidence can be associated with the results when a larger fraction of the reported interactions are validated), while the second one directly assays the reproducibility of the HT experiment. We carried out a comprehensive re-curation for all HT experiments included in HINT. Only those HT experiments that satisfy have a validation rate of >50% and a recuration rate of >75% are included in HINT. A list of all recurated HT experiments for human and *S. cerevisiae* can be found in Supplementary Tables 4 and 5. The validation

and retest rates for each of them can be found in Supplementary Tables 6 and 7.

4) On the other hand, since it is impossible to manually check all small-scale studies, we require two independent publications to report the same interaction for it to be included in our dataset. This is because, although interactions from dedicated small-scale studies are often of high quality and repeated multiple times in the literature, the fact that others do not report an interaction from a small-scale experiment raises the question of whether it is reproducible. In fact, many of these interactions supported by only one publication were not produced by dedicated experiments and were often not even mentioned in the paper. For example, Subba Rao et al [7] present a “two-hybrid-based analysis of protein-protein interactions of the yeast multidrug resistance protein, Pdr5p”. In the paper, Figure 2 shows a very weak interaction between Pdr5 and Pry3 (Supplementary Figure 5). This interaction is neither the focus of the paper, nor have the authors mentioned it anywhere in the text. No verification or validation is available to support the interaction. This is a typical example of an interaction from a small-scale study that is low-quality/erroneous and is thus not obtained by any other study. HINT aims to eliminate such interactions.

5) More importantly, it has been shown experimentally such interactions are indeed of low quality [5, 6].

References:

1. Mewes HW, Ruepp A, Theis F, Rattei T, Walter M, Frishman D, Suhre K, Spannagl M, Mayer KF, Stumpflen V *et al*: **MIPS: curated databases and comprehensive secondary data resources in 2010**. *Nucleic Acids Res* 2011, **39**(Database issue):D220-224.
2. Hu Z, Hung JH, Wang Y, Chang YC, Huang CL, Huyck M, DeLisi C: **VisANT 3.5: multi-scale network visualization, analysis and inference based on the gene ontology**. *Nucleic Acids Res* 2009, **37**(Web Server issue):W115-121.
3. Hermjakob H, Montecchi-Palazzi L, Bader G, Wojcik J, Salwinski L, Ceol A, Moore S, Orchard S, Sarkans U, von Mering C *et al*: **The HUPO PSI's molecular interaction format--a community standard for the representation of protein interaction data**. *Nat Biotechnol* 2004, **22**(2):177-183.
4. Tarassov K, Messier V, Landry CR, Radinovic S, Serna Molina MM, Shames I, Malitskaya Y, Vogel J, Bussey H, Michnick SW: **An in vivo map of the yeast protein interactome**. *Science* 2008, **320**(5882):1465-1470.

5. Venkatesan K, Rual JF, Vazquez A, Stelzl U, Lemmens I, Hirozane-Kishikawa T, Hao T, Zenkner M, Xin X, Goh KI *et al*: **An empirical framework for binary interactome mapping**. *Nat Methods* 2009, **6**(1):83-90.
6. Yu H, Braun P, Yildirim MA, Lemmens I, Venkatesan K, Sahalie J, Hirozane-Kishikawa T, Gebreab F, Li N, Simonis N *et al*: **High-quality binary protein interaction map of the yeast interactome network**. *Science* 2008, **322**(5898):104-110.
7. Subba Rao G, Bachhawat AK, Gupta CM: **Two-hybrid-based analysis of protein-protein interactions of the yeast multidrug resistance protein, Pdr5p**. *Funct Integr Genomics* 2002, **1**(6):357-366.
