# Supplementary material for: HINT: High-quality protein interactomes and their applications in understanding human disease
Source: BMC Syst Biol. 2012 Jul 30;6:92. doi: 10.1186/1752-0509-6-92 (PMC3483187; doi:10.1186/1752-0509-6-92)
Supplement: Additional file 2 — Binary and co-complex interaction networks in S. pombe. [file 1752-0509-6-92-S2.pdf]

Binary Interactions	
**Experimental Evidence**	**PSI-MI Evidence Code**
array technology	0008
beta galactosidase complementation	0010
beta lactamase complementation	0011
bioluminescence resonance energy transfer	0012
adenylate cyclase complementation	0014
circular dichroism	0016
classical fluorescence spectroscopy	0017
two hybrid	0018
coimmunoprecipitation	0019
transmission electron microscopy	0020
cosedimentation	0027
cosedimentation in solution	0028
cosedimentation through density gradient	0029
cross-linking study	0030
protein cross-linking with a bifunctional reagent	0031
dynamic light scattering	0038
electron microscopy	0040
electron paramagnetic resonance	0042
far western blotting	0047
filamentous phage display	0048
filter binding	0049
fluorescence correlation spectroscopy	0052
fluorescence polarization spectroscopy	0053
fluorescence-activated cell sorting	0054
fluorescent resonance energy transfer	0055
isothermal titration calorimetry	0065
lambda phage display	0066
light scattering	0067
molecular sieving	0071
nuclear magnetic resonance	0077
peptide array	0081
phage display	0084
protein array	0089
protein complementation assay	0090
chromatography technology	0091
reverse ras recruitment system	0097
scintillation proyimity assay	0099
static light scattering	0104
surface plasmon resonance	0107
t7 phage display	0108
dihydrofolate reductase reconstruction	0111
ubiquitin reconstruction	0112
x-ray crystallography	0114
yeast display	0115
ion eychange chromatography	0226
reverse phase chromatography	0227

Supplementary Table 1

green fluorescence protein complementation assay	0229
mammalian protein protein interaction trap	0231
transcriptional complementation assay	0232
blue native page	0276
ley-a dimerization assay	0369
toy-r dimerization assay	0370
two hybrid array	0397
two hybrid pooling approach	0398
two hybrid fragment pooling approach	0399
comigration in non denaturing gel electrophoresis	0404
competition binding	0405
deacetylase assay	0406
electron tomography	0410
enzyme linked immunosorbent assay	0411
enzymatic study	0415
fluorescence microscopy	0416
kinase homogeneous time resolved fluorescence	0420
in-gel kinase assay	0423
protein kinase assay	0424
phosphatase assay	0434
protease assay	0435
saturation binding	0440
homogeneous time resolved fluorescence	0510
methyltransferase assay	0515
methyltransferase radiometric assay	0516
enzymatic footprinting	0605
lambda repressor two hybrid	0655
antibody array	0678
reverse two hybrid	0726
gal4 vp16 complementation	0728
luminescence based mammalian interactome mapping	0729
comigration in gel electrophoresis	0807
comigration in sds page	0808
bimolecular fluorescence complementation	0809
y-ray fiber diffraction	0825
y ray scattering	0826
phosphotransfer assay	0841
immunodepleted coimmunoprecipitation	0858
intermolecular force	0859
demethylase assay	0870
atomic force microscopy	0872
acetylation assay	0889
surface plasmon resonance array	0921
polymerization	0953

Co-complex Associations	
**Experimental Evidence**	**PSI-MI Evidence Code**
affinity chromatography technology	0004
anti bait coimmunoprecipitation	0006
anti tag coimmunoprecipitation	0007
mass spectrometry studies of complexes	0069
pull down	0096
affinity technology	0400
tandem affinity purification	0676

Supplementary Table 1
