# Supplementary material for: HINT: High-quality protein interactomes and their applications in understanding human disease
Source: BMC Syst Biol. 2012 Jul 30;6:92. doi: 10.1186/1752-0509-6-92 (PMC3483187; doi:10.1186/1752-0509-6-92)
Supplement: Additional file 3 — Binary protein-protein interactions in human – HT studies. [file 1752-0509-6-92-S3.pdf]

Binary Interactions		
MIPS Evidence Code	MIPS Description	Corresponding PSI-MI Code
902.01.01.02.01.01	co-immunoprecipitation	0019
902.01.01.02.01.03	centrifugation	0027
902.01.01.02.01.05.01	cross linking, chemical	0031
902.01.01.02.01.05.02	cross linking, UV	0430
902.01.01.02.01.06	in vitro reconstitution	0492
902.01.01.02.01.07	two hybrid	0018
902.01.01.02.01.08	overlay	0047
902.01.01.02.01.09.01	FRET	0055
902.01.01.02.01.09.02	scintillation proximity assay	0099
902.01.01.02.01.10	surface plasmon resonance	0107
902.01.01.02.01.11	phage display	0084
902.01.01.02.01.13.01	electron microscopy	0040
902.01.01.02.01.13.02	NMR	0077

Co-complex Associations		
MIPS Evidence Code	MIPS Description	Corresponding PSI-MI Code
902.01.01.02.01	physical	0013
902.01.01.02.01.01.02	epitope tag co-ip	0007
902.01.01.02.01.02	affinity chromatography	0004
902.01.01.02.01.02.01	affinity chromatography, native	0004
902.01.01.02.01.02.02	affinity tag chromatography	0004

Supplementary Table 2
