# Supplementary material for: HINT: High-quality protein interactomes and their applications in understanding human disease
Source: BMC Syst Biol. 2012 Jul 30;6:92. doi: 10.1186/1752-0509-6-92 (PMC3483187; doi:10.1186/1752-0509-6-92)
Supplement: Additional file 4 — Binary protein-protein interactions in S. cerevisiae – HT studies. [file 1752-0509-6-92-S4.pdf]

Binary Interactions		
VisAnt Evidence Code	VisAnt Description	Corresponding PSI-MI Code
M0010	Co-immunoprecipitation	0019
M0011	Co-sedimentation	0027
M0012	Competition binding	0405
M0013	Copurification	0025
M0014	Cross-linking studies	0030
M0015	Electron microscopy	0040
M0018	Molecular sieving	0071
M0021	Western blot	0113
M0024	Immunoprecipitation	0019
M0026	In vitro binding	0492
M0029	Monoclonal antibody	0671
M0032	Sizing Column	0071
M0033	Cosedimentation	0027
M0034	Two-hybrid	0018
M0035	X-ray	0114
M0049	Surface plasmon resonance	0921
M0050	Phage display	0084
M0051	ELISA	0411
M0052	Fluorescence technology	0051
M0053	Filter binding	0928
M0060	Far western	0047
M0061	Resonance energy transfer	0055
M0062	Electron microscopy	0040
M0066	Enzymatic study	0415
M0068	Protein array	0089
M0069	Protein complementation assay	0090
M0070	NMR	0077
M0071	X-ray crystallography	0114
M0079	Co-fractionation	0027
M0085	Chromatography	0091
M0092	Peptide array	0081
M0095	Protein kinase assay	0424
M0096	Blue native PAGE	0276
M0097	Comigration in gel electophoresis	0404
M0100	Ubiquitin reconstruction	0112
M0101	Phosphatase assay	0434
M0103	Isothermal titration calorimetry	0065

Co-complex Associations		
VisAnt Evidence Code	VisAnt Description	Corresponding PSI-MI Code
M0006	Affinity column	0400
M0028	Mass spectrometry of complex	0069
M0044	Affinity precipitation	0400
M0045	Affinity technology	0400
M0065	Anti-tag co-IP	0007
M0067	Pull down	0096

Supplementary Table 3

M0074	Reconstituted complex	0069
M0088	Tandem affinity purification	0676
M0089	Anti-bait Co-IP	0006
M5001	Tandem affinity mass spectrometry	0032
