# Supplementary material for: HINT: High-quality protein interactomes and their applications in understanding human disease
Source: BMC Syst Biol. 2012 Jul 30;6:92. doi: 10.1186/1752-0509-6-92 (PMC3483187; doi:10.1186/1752-0509-6-92)
Supplement: Additional file 5 — Validation and retest rates for binary protein-protein interactions in human – HT studies. [file 1752-0509-6-92-S5.pdf]

Binary protein-protein interactions in human - HT studies						
Pubmed id	Title	Author & Citation	Number of interactions reported	Technique used	Included	Reason for exclusion
12421765	Protein-protein interactions between large proteins: two-hybrid screening using a functionally classified library composed of long cDNAs.	Nakayama et al, Genome Research 2002, 12(11):1773-84	116	Yeast two-hybrid	no	Does not satisfy the validation and retest rate cutoffs
12805554	Comprehensive identification of human bZIP interactions with coiled-coil arrays.	Newman et al, Science 2003, 300(5628):2097-101	280	Array technology	yes	
14667819	Analysis of a high-throughput yeast two-hybrid system and its use to predict the function of intracellular proteins encoded within the human MHC class III region.	Lehner et al, Genomics 2004, 83(1):153-67	110	Yeast two-hybrid	yes	
14743216	A physical and functional map of the human TNF-alpha/NF-kappa B signal transduction pathway.	Bouwmeester et al, Nature Cell Biology 2004, 6(2):97-105	128	Affinity purification followed by mass spectrometry	no	Uses AP/MS, included in the co-complex dataset instead
14988562	Control of pancreas and liver gene expression by HNF transcription factors.	Odom et al, Science 2004, 303(5662):1378-81	2467	Chromatin immunoprecipitation	no	Reports protein-DNA, not protein-protein interactions
15163412	RNA and RNA binding proteins participate in early stages of cell spreading through spreading initiation centers.	de Hoog et al, Cell 2004, 117(5):649-62	330	Affinity purification followed by mass spectrometry	no	Uses mass spectometry, included in the co-complex dataset instead
15231747	A protein interaction framework for human mRNA degradation.	Lehner et al, Genome Research 2004, 14(7):1315-23	263	Yeast two-hybrid	yes	
15231748	Functional proteomics mapping of a human signaling pathway.	Colland et al, Genome Research 2004, 14(7):1324-32	755	Yeast two-hybrid	yes	
15232106	Self-assembling protein microarrays.	Ramachandran et al, Science 2004, 305(5680):86-90	103	Protein microarray	yes	
15324660	Proteomic, functional, and domain-based analysis of in vivo 14-3-3 binding proteins involved in cytoskeletal regulation and cellular organization.	Jin et al, Current Biology 2004, 14(16):1436-50	298	Affinity purification followed by mass spectrometry	no	Uses mass spectometry, included in the co-complex dataset instead
15383276	A protein interaction network links GIT1, an enhancer of huntingtin aggregation, to Huntington's disease.	Goehler et al, Molecular Cell 2004, 15(6):853-65	154	Yeast two-hybrid	yes	
15604093	Automated yeast two-hybrid screening for nuclear receptor-interacting proteins.	Albers et al, Molecular & Cellular Proteomics 2005, 4(2):205-13	377	Yeast two-hybrid	yes	
15761153	High-throughput mapping of a dynamic signaling network in mammalian cells.	Barrios-Rodiles et al, Science 2005, 307(5715):1621-5	429	Luminescence-based mammalian interactome mapping	no	Does not satisfy the validation and retest rate cutoffs
16169070	A human protein-protein interaction network: a resource for annotating the proteome.	Stelzl et al, Cell 2005, 122(6):957-68	2480	Yeast two-hybrid	yes	
16189514	Towards a proteome-scale map of the human protein-protein interaction network.	Rual et al, Nature 2005, 437(7062):1173-8	2618	Yeast two-hybrid	yes	
16273093	A quantitative protein interaction network for the ErbB receptors using protein microarrays.	Jones et al, Nature 2006, 439(7073):168-74	160	Protein microarray	yes	

Supplementary Table 4

16713569	A protein-protein interaction network for human inherited ataxias and disorders of Purkinje cell degeneration.	Lim et al, Cell 2006, 125(4):801-14	770	Yeast two-hybrid	no	Does not satisfy the validation and retest rate cutoffs
17043677	Disrupted in Schizophrenia 1 Interactome: evidence for the close connectivity of risk genes and a potential synaptic basis for schizophrenia.	Camargo et al, Molecular Psychiatry 2007, 12(1):74-86	264	Yeast two-hybrid	yes	
17474147	Systematic identification of SH3 domain-mediated human protein-protein interactions by peptide array target screening.	Wu et al, Proteomics 2007, 7(11):1775-85	958	Peptide array target screening	yes	
18624398	Protein interaction data set highlighted with human Ras-MAPK/PI3K signaling pathways.	Wang et al, Journal of Proteome Research 2008, 7(9):3879-89	200	Yeast two-hybrid	no	Does not satisfy the validation and retest rate cutoffs
18654987	Identification of multi-SH3 domain-containing protein interactome in pancreatic cancer: a yeast two-hybrid approach.	Thalappilly et al, Proteomics 2008, 8(15):3071-81	147	Yeast two-hybrid	no	Does not satisfy the validation and retest rate cutoffs
19549727	Analysis of the human E2 ubiquitin conjugating enzyme protein interaction network.	Markson et al, Genome Research 2009, 19(10):1905-11	557	Yeast two-hybrid	no	Does not satisfy the validation and retest rate cutoffs
19690564	A comprehensive framework of E2-RING E3 interactions of the human ubiquitin-proteasome system.	van Wijk et al, Molecular Systems Biology 2009, 5:295	474	Yeast two-hybrid	yes	
20211142	An atlas of combinatorial transcriptional regulation in mouse and man.	Ravasi et al, Cell 2010, 140(5):744-52	672	Mammalian two-hybrid	no	Does not satisfy the validation and retest rate cutoffs
20936779	A human MAP kinase interactome.	Bandyopadhyay et al, Nature Methods 2010, 7(10):801-5	636	Yeast two-hybrid	yes	
21078624	Comparison of an expanded ataxia interactome with patient medical records reveals a relationship between macular degeneration and ataxia.	Kahle et al, Human Molecular Genetics 2011, 20(3):510-27	134	Yeast two-hybrid	yes	
21163940	Interactome mapping suggests new mechanistic details underlying Alzheimer's disease.	Soler-López et al, Genome Research 2011, 21(3):364-76	200	Yeast two-hybrid	yes	
21182203	A large set of estrogen receptor β-interacting proteins identified by tandem affinity purification in hormone-responsive human breast cancer cell nuclei.	Nassa et al, Proteomics 2011, 11(1):159-65	140	Tandem affinity purification	no	Uses mass spectometry, included in the co-complex dataset instead
21182205	Identification of proteins associated with ligand-activated estrogen receptor α in human breast cancer cell nuclei by tandem affinity purification and nano LC-MS/MS.	Tarallo et al, Proteomics 2011, 11(1):172-9	147	Affinity purification followed by mass spectrometry	no	Uses mass spectometry, included in the co-complex dataset instead
21516116	Next-generation sequencing to generate interactome datasets.	Yu et al, Nature Methods 2011, 8(6):478-80	589	Yeast two-hybrid	yes	
21900206	A directed protein interaction network for investigating intracellular signal transduction.	Vinayagam et al, Science Signaling 2001, 4(189):rs8	2626	Yeast two-hybrid	yes	

Supplementary Table 4
