# Supplementary material for: HINT: High-quality protein interactomes and their applications in understanding human disease
Source: BMC Syst Biol. 2012 Jul 30;6:92. doi: 10.1186/1752-0509-6-92 (PMC3483187; doi:10.1186/1752-0509-6-92)
Supplement: Additional file 6 — Validation and retest rates for binary protein-protein interactions in S. cerevisiae – HT studies. [file 1752-0509-6-92-S6.pdf]

Binary protein-protein interactions in *S. cerevisiae* - HT studies						
**Pubmed id**	**Title**	**Author & Citation**	**Number of interactions reported**	**Technique used**	**Included**	**Reason for exclusion**
10655498	Toward a protein-protein interaction map of the budding yeast: A comprehensive system to examine two-hybrid interactions in all possible combinations between the yeast proteins.	Ito et al, Proceedings of the National Academy of Sciences 2000, 97(3):1143-7	183	Yeast two-hybrid	no	Only core dataset included as the non-core dataset does not pass the validation and retest rate thresholds
10688190	A comprehensive analysis of protein-protein interactions in Saccharomyces cerevisiae.	Uetz et al, Nature 2000, 403(6770):623-7	957	Yeast two-hybrid	yes	
10900456	Genome-wide protein interaction screens reveal functional networks involving Sm-like proteins.	Fromont-Racine et al, Yeast 2000, 17(2):95-110	112	Yeast two-hybrid	no	Does not satisfy the validation and retest rate cutoffs
11087867	A computationally directed screen identifying interacting coiled coils from Saccharomyces cerevisiae.	Newman et al, Proceedings of the National Academy of Sciences 2000, 97(24):13203-8	159	Yeast two-hybrid	yes	
11283351	A comprehensive two-hybrid analysis to explore the yeast protein interactome.	Ito et al, Proceedings of the National Academy of Sciences 2001, 10;98(8):4569-74	4549	Yeast two-hybrid	no	Only core dataset included as the non-core dataset does not pass the validation and retest rate thresholds
11489916	A protein interaction map for cell polarity development.	Drees et al, The Journal of Cell Biology 2001, 154(3):549-71	191	Yeast two-hybrid	yes	
11743162	A combined enoperimental and computational strategy to define protein interaction networks for peptide recognition modules.	Tong et al, Science 2002, 295(5553):321-4	232	Yeast two-hybrid	no	Does not satisfy the validation and retest rate cutoffs
14574415	Targets of the cyclin-dependent kinase Cdk1.	Ubersax et al, Nature 2003, 425(6960):859-64	184	Protein kinase assay	yes	
14690591	Assigning function to yeast proteins by integration of technologies.	Hazbun et al, Molecular Cell 2003, 12(6):1353-65	2501	Affinity purification followed by mass spectometry	no	Uses AP/MS, included in the co-complex dataset instead
14737190	Protein interaction networks by proteome peptide scanning.	Landgraf et al, PloS Biology 2004, 2(1):E14	183	Phage display	yes	
15879519	A two-hybrid screen of the yeast proteome for Hsp90 interactors uncovers a novel Hsp90 chaperone requirement in the activity of a stress-activated mitogen-activated protein kinase, Slt2p (Mpk1p).	Millson et al, Eukaryotic Cell 2005, 4(5):849-60	124	Yeast two-hybrid	no	Does not satisfy the validation and retest rate cutoffs

Supplementary Table 5

16093310	Large-scale identification of yeast integral membrane protein interactions.	Miller et al Proceedings of the National Academy of Sciences 2005, 102(34):12123-8	1977	Yeast two-hybrid	yes	
16319894	Global analysis of protein phosphorylation in yeast.	Ptacek et al, Nature 2005, 438(7068):679-84	4023	Proteome chip	no	Detects phosphorylation, not direct protein-protein interactions
16606443	Comparative analysis of Saccharomyces cerevisiae WW domains and their interacting proteins.	Hesselberth et al, Genome Biology 2006, 7(4):R30	517	Protein microarray	yes	
17634282	A protein interaction map of the mitotic spindle.	Wong et al, Molecular Biology of the Cell 2007, 18(10):3800-9	585	Yeast two-hybrid	yes	
18467557	An in vivo map of the yeast protein interactome.	Tarassov et al, Science 2008, 320(5882):1465-70	2770	Dihydrofolate reductase protein complementation assay	no	Included as co-complex associations as reported in the manuscript
18719252	High-quality binary protein interaction map of the yeast interactome network.	Yu et al, Science 2008, 322(5898):104-10	1778	Yeast two-hybrid	yes	
19841731	Bayesian modeling of the yeast SH3 domain interactome predicts spatiotemporal dynamics of endocytosis proteins.	Tonikian et al, PloS Biology 2009, 7(10):e1000218	957	Peptide array	yes	
21118957	A constraint network of interactions: protein-protein interaction analysis of the yeast type II phosphatase Ptc1p and its adaptor protein Nbp2p.	Hruby et al, Journal of Cell Science 2011, 124(Pt 1):35-46	129	Protein complementation assay	no	Does not satisfy the validation and retest rate cutoffs
21748599	Interactomic study on interaction between lipid droplets and mitochondria.	Pu et al, Protein Cell 2011, 2(6):487-96.	116	Bimolecular fluorescence complementation	no	Does not satisfy the validation and retest rate cutoffs
9207794	Toward a functional analysis of the yeast genome through enohaustive two-hybrid screens.	Fromont-Racine et al, Nature Genetics 1997, 16(3):277-82	160	Yeast two-hybrid	no	Does not satisfy the validation and retest rate cutoffs

Supplementary Table 5
