# Supplementary material for: HINT: High-quality protein interactomes and their applications in understanding human disease
Source: BMC Syst Biol. 2012 Jul 30;6:92. doi: 10.1186/1752-0509-6-92 (PMC3483187; doi:10.1186/1752-0509-6-92)
Supplement: Additional file 7 — Example of an interaction from a small-scale study with low-quality supporting evidence. [file 1752-0509-6-92-S7.pdf]

Validation and retest rates for binary protein-protein interactions in human - HT studies					
**Pubmed id**	**Number of interactions reported**	**Number of interactions validated**	**Validation rate**	**Number of validated interactions that tested positive**	**Retest rate**
12421765	116	84	72.41	42	50
12805554	280	280	100	280	100
14667819	110	103	93.64	103	100
14743216	128	NA	NA	NA	NA
14988562	2467	NA	NA	NA	NA
15163412	330	NA	NA	NA	NA
15231747	263	263	100	247	93.92
15231748	755	755	100	576	76.29
15232106	103	83	80.58	66	79.52
15324660	298	NA	NA	NA	NA
15383276	186	186	100	186	100
15604093	377	377	100	377	100
15761153	429	0	0	0	0
16169070	2600	2600	100	2600	100
16189514	2618	*	*	*	*
16273093	160	160	100	160	100
16713569	770	75	9.74	62	82.67
17043677	158	158	100	158	100
17474147	958	958	100	958	100
18624398	200	16	8	15	93.75
18654987	147	5	3.40	5	100
19549727	557	84	15.08	78	92.86
19690564	474	474	100	474	100
20211142	762	34	4.46	18	52.94
20936779	641	641	100	641	100
21078624	118	61	51.69	46.00	75.41
21163940	200	200	100	200	100
21182203	140	NA	NA	NA	NA
21182205	147	NA	NA	NA	NA
21516116	1166	**	**	**	**
21900206	2626	2626	100	2626	100
** The entire dataset was subject to a comprehensive quality assessment using orthogonal assays as described in Rual et al Nature 2005. The precision is estimated to be ~ 78%.					
** The entire dataset was subject to a comprehensive quality assessment using several orthogonal assays as described in Yu et al Nature Methods 2011, Venkatesan et al Nature Methods 2009 & Yu et al Science 2008. The authors found that the precision of this dataset is comparable to the benchmark established in Yu et al Science 2008 i.e. ~ 94%					
NA refers to experiments that have not validated any interactions or use techniques that are inappropriate to detect binary interactions (please refer to Supplementary Table 5).					

Supplementary Table 6
