# Supplementary material for: HINT: High-quality protein interactomes and their applications in understanding human disease
Source: BMC Syst Biol. 2012 Jul 30;6:92. doi: 10.1186/1752-0509-6-92 (PMC3483187; doi:10.1186/1752-0509-6-92)
Supplement: Additional file 8 — Overlaps between binary and co-complex interaction, HT and LC interaction networks in human and S. cerevisiae. [file 1752-0509-6-92-S8.pdf]

Validation and retest rates for binary protein-protein interactions in *S. cerevisiae* - HT studies					
**Pubmed id**	**Number of interactions reported**	**Number of interactions validated**	**Validation rate**	**Number of validated interactions that tested positive**	**Retest rate**
10655498	183	*	*	*	*
10688190	957	*	*	*	*
10900456	112	0	0	0	NA
11087867	159	159	100	159	100
11283351	4549	*	*	*	*
11489916	191	191	100	191	100
11743162	232	59	25.43	59	100
14574415	181	181	100	181	100
14690591	2501	NA	NA	NA	NA
14737190	183	183	100	183	100
15879519	124	NA	NA	NA	NA
16093310	808	808	100	808	100
16319894	4023	NA	NA	NA	NA
16606443	587	587	100	587	100
17634282	604	604	100	604	100
18467557	2770	NA	NA	NA	NA
18719252	1778	*	*	*	*
19841731	749	749	100	593	79.17
21118957	129	0	0	0	NA
21748599	116	0	0	0	NA
9207794	160	0	0	0	NA
* The Uetz, Ito and Yu datasets were subject to a comprehensive quality assessment using several orthogonal assays in Yu et al Science 2008. The authors found that for the Uetz, Ito core and Yu datasets, the precision is ~ 94%					
NA refers to experiments that have not validated any interactions or use techniques that are inappropriate to detect binary interactions (please refer to Supplementary Table 5).					

Supplementary Table 7
