# Supplementary figures and images for: HINT: High-quality protein interactomes and their applications in understanding human disease
Source: BMC Syst Biol. 2012 Jul 30;6:92. doi: 10.1186/1752-0509-6-92 (PMC3483187; doi:10.1186/1752-0509-6-92)

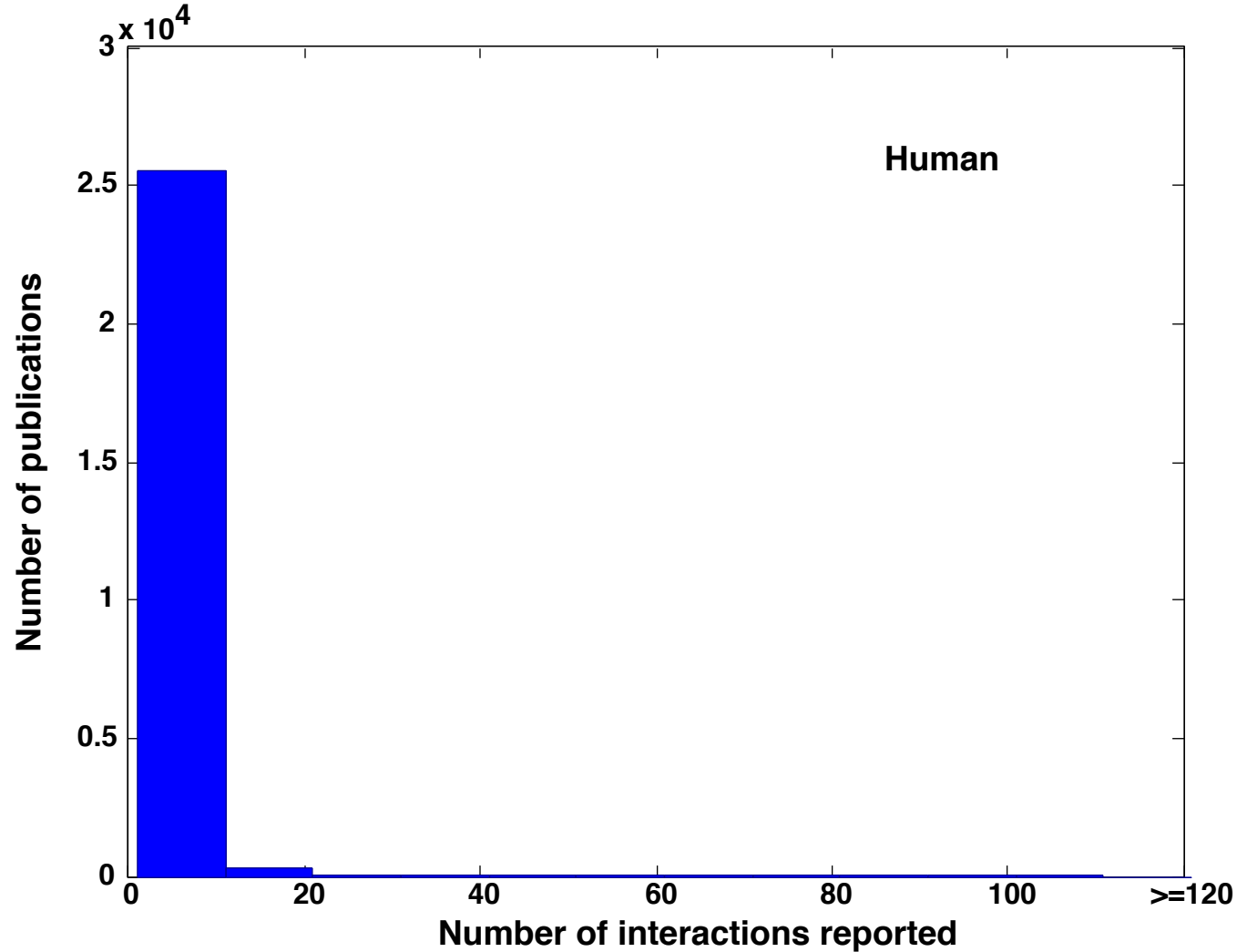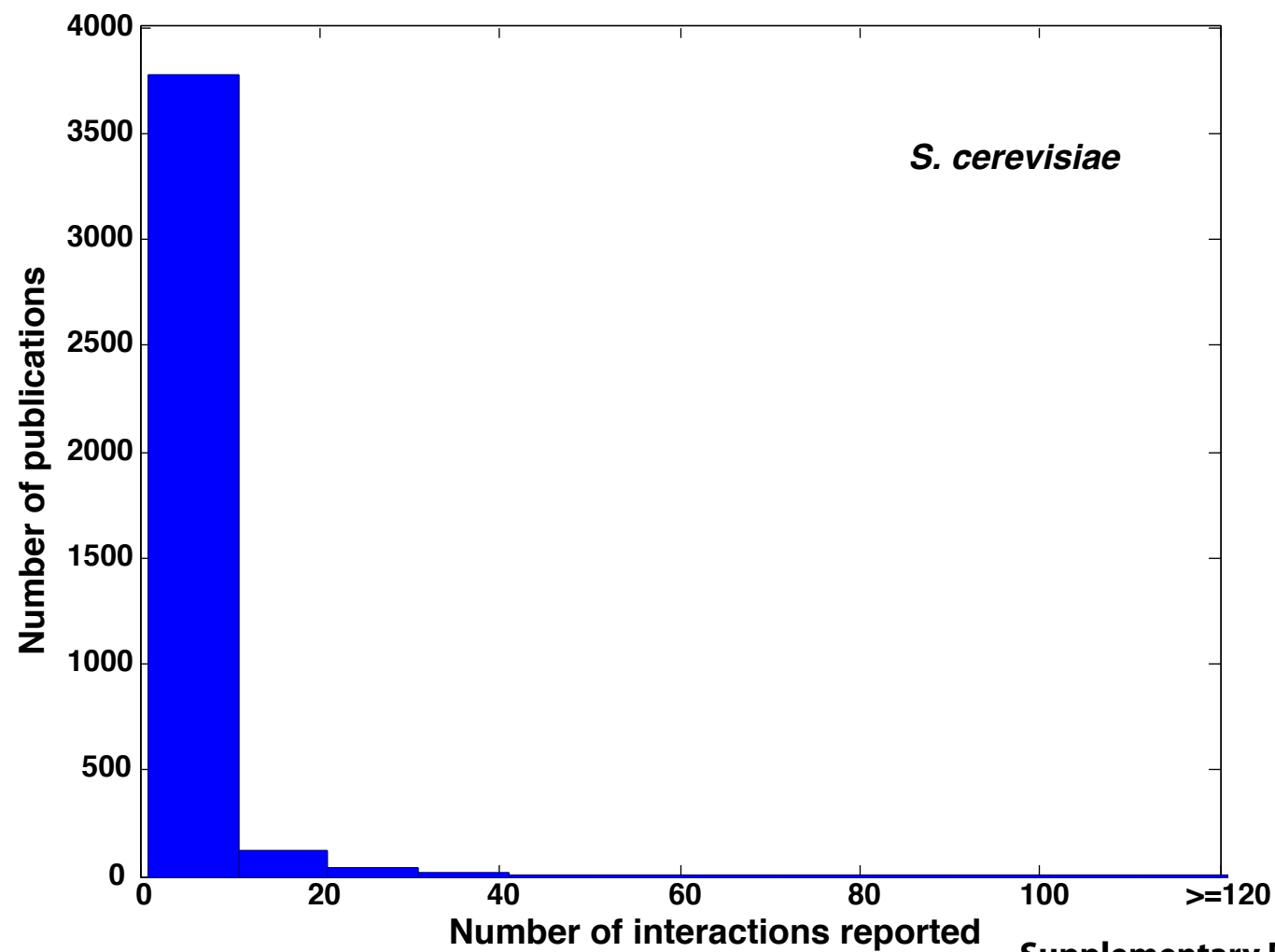

Supplement: Additional file 9 — Clustering coefficient and edge betweenness for binary and co-complex networks in human and S. cerevisiae. [file 1752-0509-6-92-S9.pdf]

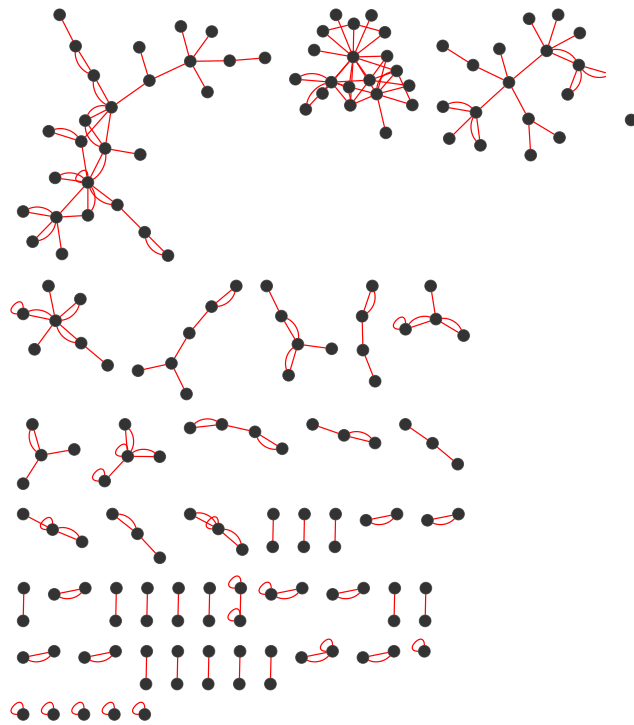

***S. pombe* binary interactome**

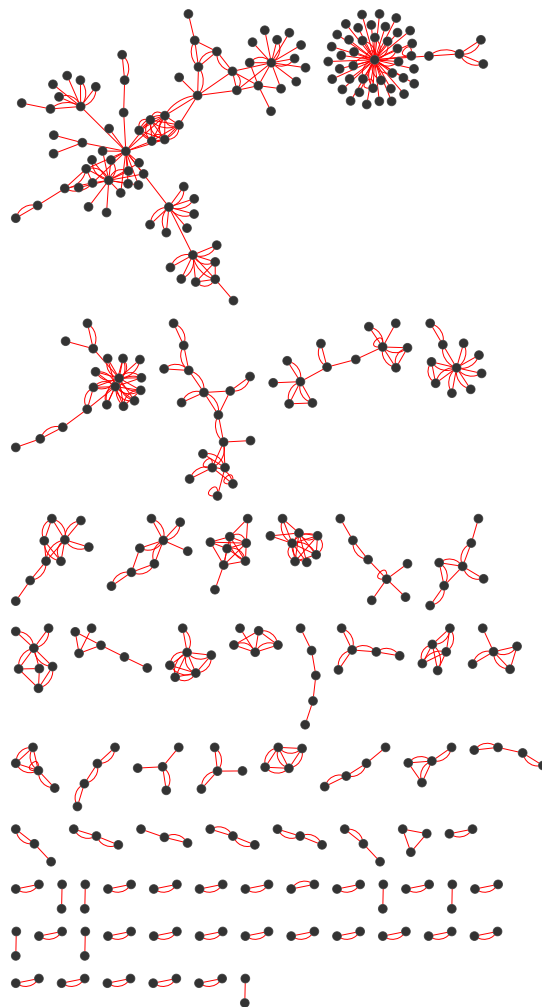

***S. pombe* co-complex interactome**

Supplement: Additional file 10 — List of PSI-MI evidence codes used to classify binary interactions and co-complex associations. [file 1752-0509-6-92-S10.pdf]

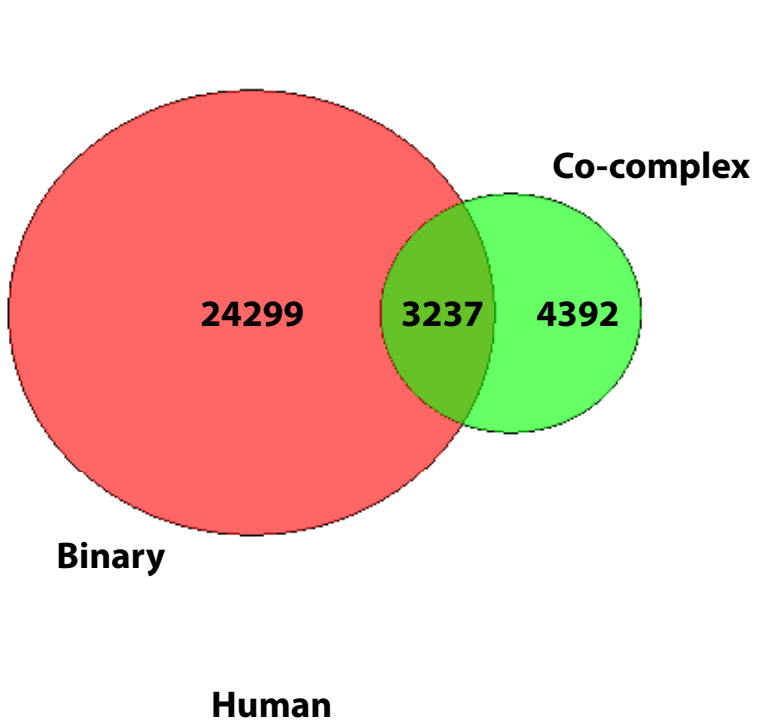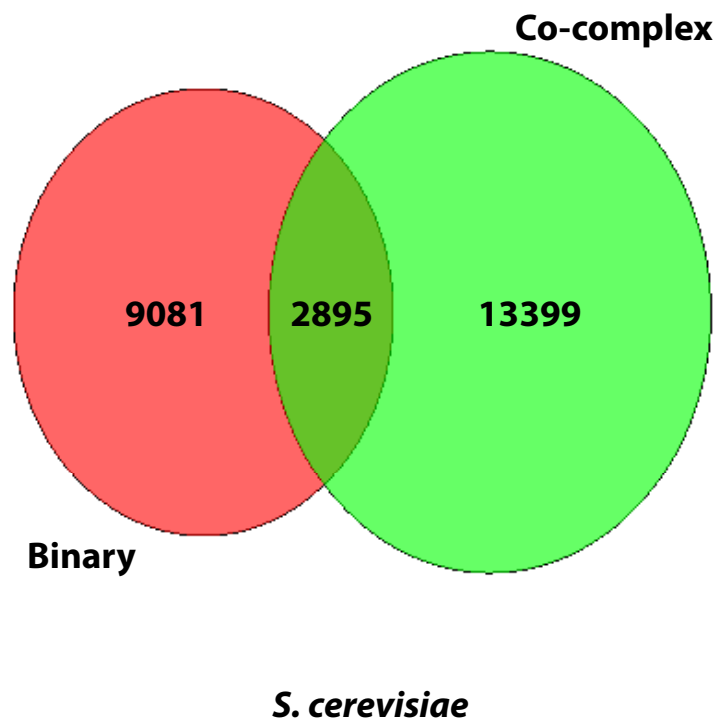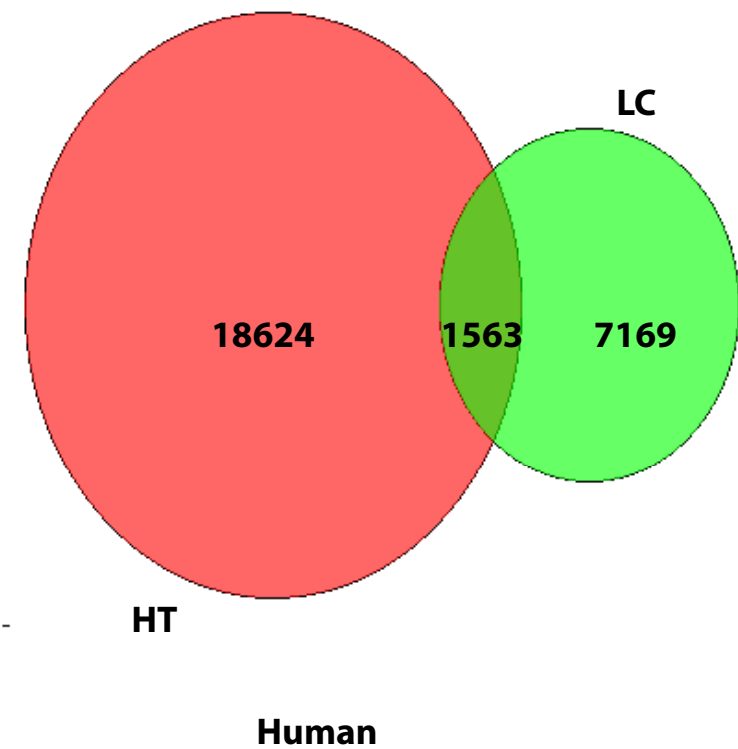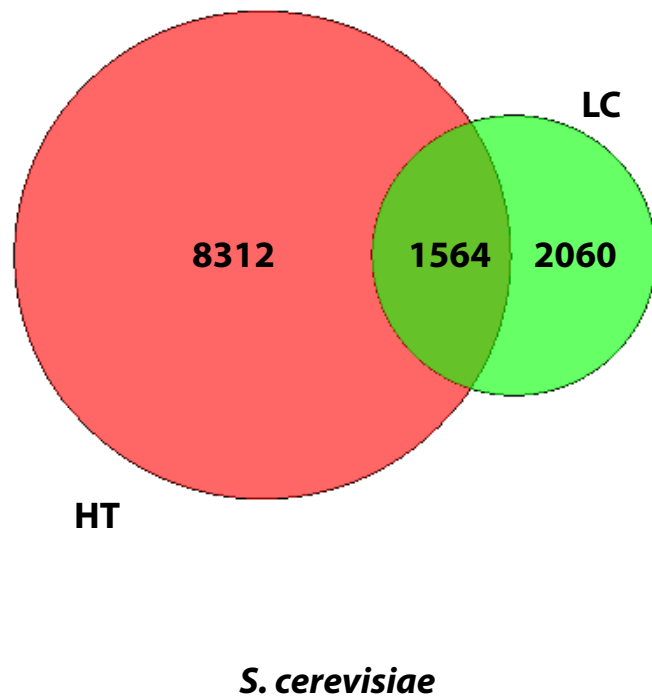

Supplement: Additional file 11 — Mapping used to convert MIPS evidence codes to PSI-MI evidence codes. [file 1752-0509-6-92-S11.pdf]

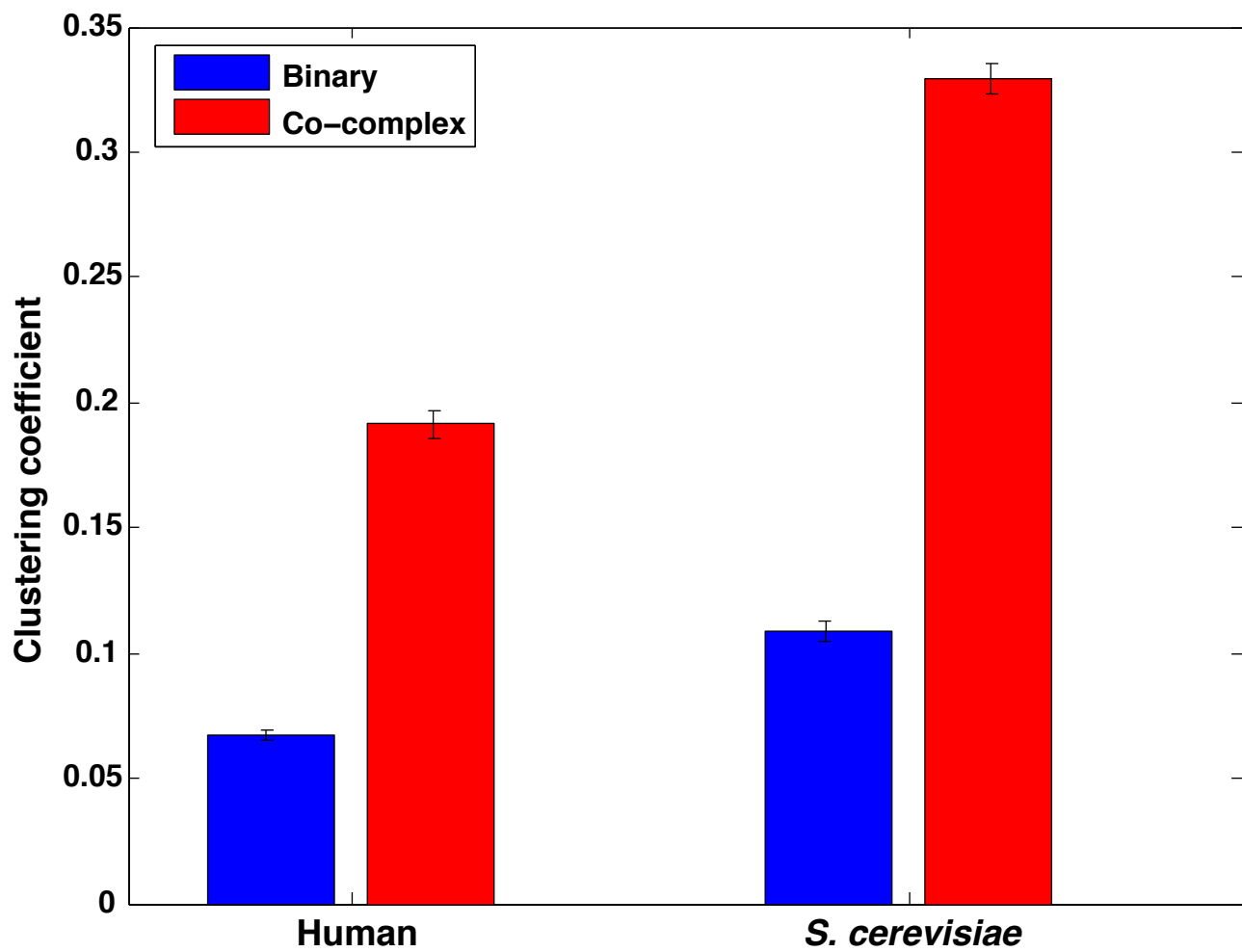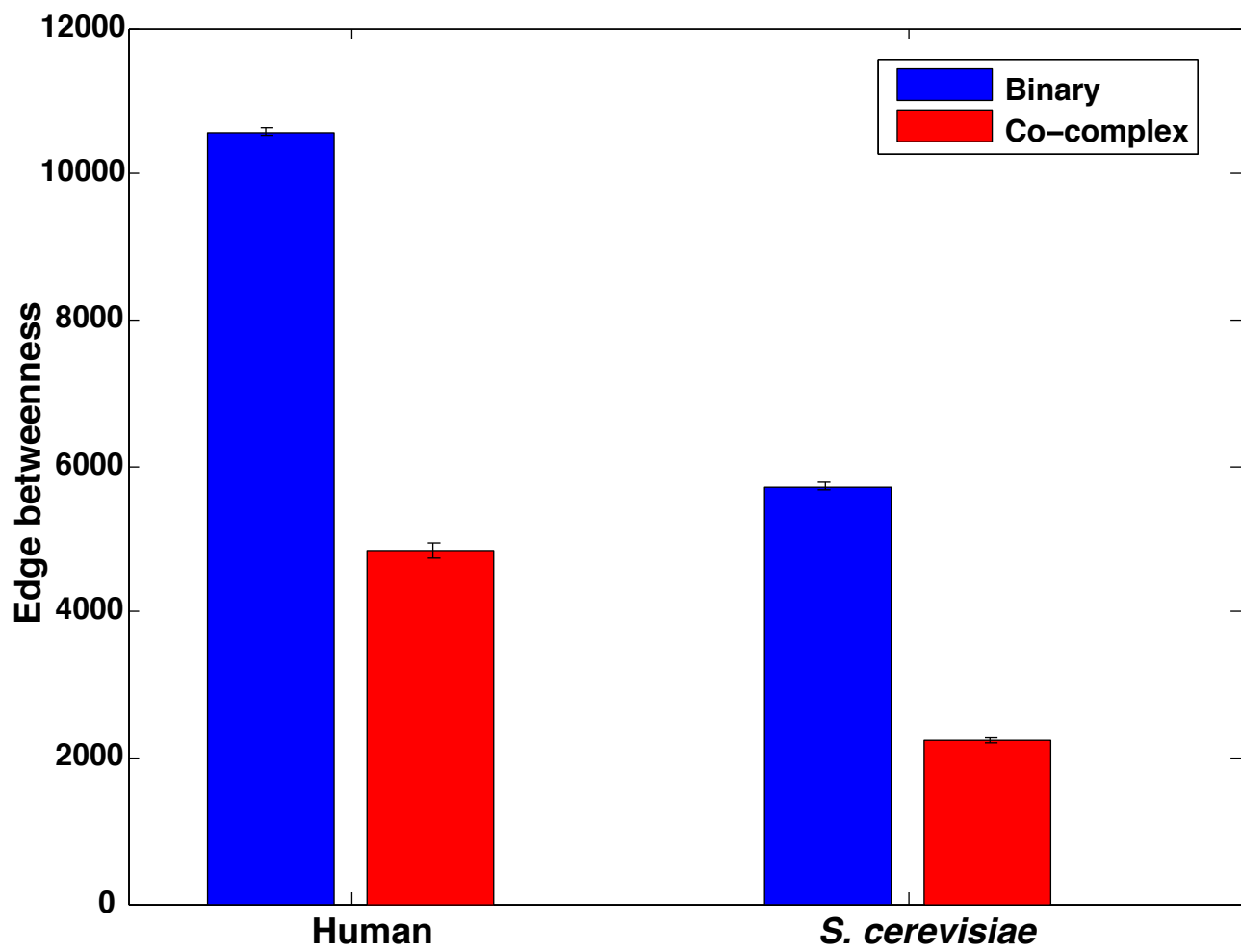

Supplementary Figure 4

Supplement: Additional file 12 — Mapping used to convert VisAnt evidence codes to PSI-MI evidence codes. [file 1752-0509-6-92-S12.pdf]
