# Supplementary material for: HINT: High-quality protein interactomes and their applications in understanding human disease
Source: BMC Syst Biol. 2012 Jul 30;6:92. doi: 10.1186/1752-0509-6-92 (PMC3483187; doi:10.1186/1752-0509-6-92)
Supplement: Additional file 13 — Description of database-specific filtering techniques. [file 1752-0509-6-92-S13.pdf]

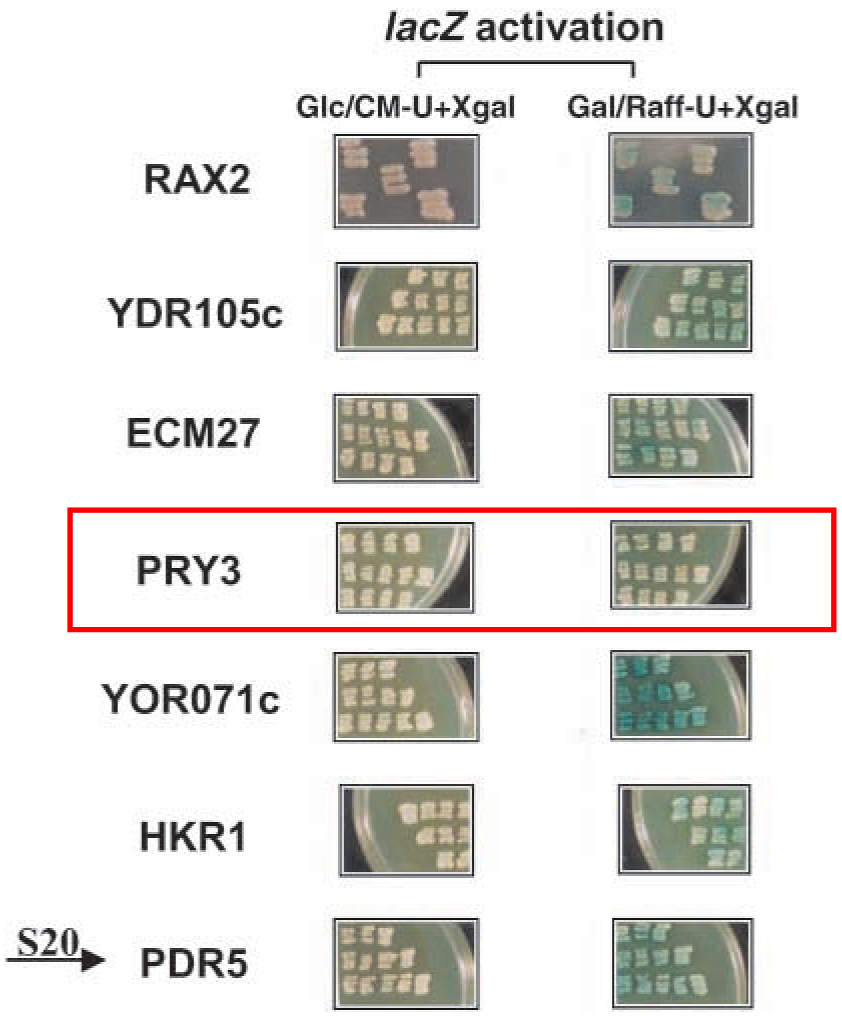

Adapted from Figure 2 of Subba Rao et al (PMID: 11957110)

Interactions of Prdp5 with different proteins tested using Y2H.
The highlighted box shows that evidence for a Pdrp5-Pry3 interaction is extremely weak.

Supplementary Figure 5
